# Hyperinflammation by Human Macrophages Induced by SARS‐CoV‐2 Anti‐Spike IgG Is Dependent on Glucose and Fatty Acid Metabolism

**DOI:** 10.1002/eji.70087

**Published:** 2025-12-19

**Authors:** Chiara E. Geyer, Luís Almeida, Lynn Mes, Frank Otto, W. Ashwin Mak, Graham A. Heieis, Jennifer Veth, Steven W. de Taeye, Tom G. Caniels, Tom P. L. Bijl, Marit J. van Gils, Menno de Winther, Amsterdam UMC COVID‐19 Biobank, Jan Van den Bossche, Hung‐Jen Chen, Riekelt H. Houtkooper, Bart Everts, Jeroen den Dunnen

**Affiliations:** ^1^ Center for Infection and Molecular Medicine, Amsterdam institute for Immunology and Infectious diseases, Amsterdam University Medical Center (UMC) University of Amsterdam Amsterdam The Netherlands; ^2^ Leiden University Center for Infectious Diseases Leiden University Medical Center Leiden The Netherlands; ^3^ Department of Medical Microbiology, Amsterdam institute for Immunology and Infectious diseases, Amsterdam University Medical Center (UMC) University of Amsterdam Amsterdam The Netherlands; ^4^ Department of Medical Biochemistry Experimental Vascular Biology Amsterdam Cardiovascular Sciences Amsterdam Infection and Immunity Amsterdam UMC University of Amsterdam Amsterdam The Netherlands; ^5^ Department of Molecular Cell Biology and Immunology Vrije Universiteit Amsterdam Amsterdam The Netherlands; ^6^ Laboratory Genetic Metabolic Diseases Amsterdam UMC University of Amsterdam Amsterdam The Netherlands; ^7^ Amsterdam Gastroenterology, Endocrinology, and Metabolism Amsterdam The Netherlands; ^8^ Amsterdam Cardiovascular Sciences Institute Amsterdam The Netherlands

**Keywords:** antibodies, COVID‐19, immunometabolism, macrophage

## Abstract

Severe COVID‐19 is an immunological disorder characterized by excessive immune activation following infection with SARS‐CoV‐2, which typically occurs around the time of seroconversion. Anti‐spike IgG of critically ill COVID‐19 patients induces excessive inflammation by activation of Fc gamma receptors (FcγRs) on human alveolar macrophages, leading to tissue damage, pulmonary edema, and coagulopathy. While metabolic reprogramming of immune cells is critical for the induction of inflammatory responses, still little is known about the metabolic pathways that are involved in COVID‐19‐specific hyperinflammation. In this study, we identified that anti‐spike IgG immune complexes (ICs) induce rapid metabolic reprogramming of alveolar macrophages, which is essential for the induction of inflammation. Through functional inhibition, we identified that glycolysis, fatty acid synthesis, and pentose phosphate pathway (PPP) activation are critical for anti‐spike IgG‐induced hyperinflammation. Remarkably, while excessive proinflammatory cytokine production by macrophages is critically dependent on simultaneous stimulation with viral stimuli and anti‐spike IgG complexes, we show that the required metabolic reprogramming is specifically driven by anti‐spike IgG complexes. These findings provide new insights into the metabolic pathways driving hyperinflammation by macrophages in the context of severe COVID‐19. Targeting of these pathways may reveal new possibilities to counteract pathological inflammatory responses in severe COVID‐19 and related diseases.

## Graphical abstract

Severe COVID‐19 is associated with hyperinflammation driven by anti‐spike IgG immune complexes (ICs) contributing to macrophage hyperactivation, thrombosis, and tissue damage. This study shows that anti‐spike IgG ICs induce rapid metabolic reprogramming, specifically glycolysis, fatty acid synthesis, and the pentose phosphate pathway, which is essential for the inflammatory response. Targeting these metabolic pathways may offer new therapeutic strategies for controlling excessive inflammation in severe COVID‐19.

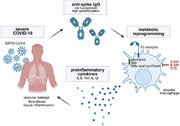

## 1 Introduction

The recent pandemic has prominently shown how airborne viruses such as SARS‐CoV‐2 can cause a significant risk to public health [1]. Even though the fast development of highly efficient vaccines has successfully dissolved the acute situation in most countries, breakthrough infections and the occurrence of new variants of concern still especially endanger risk groups such as immunocompromised, obese, elderly, or diabetic individuals [1, 2, 3, 4, 5]. It is thus crucial to further investigate the mechanisms underlying the development of severe COVID‐19 as well as to find specific drugs to treat patients experiencing severe disease progression.

Anti‐spike IgG immune complexes (ICs) play a key role in the development of severe COVID‐19 progression by activation of Fc receptors (FcRs) [6, 7, 8]. Patients experiencing severe COVID‐19 develop a pathological antibody response characterized by high titers of anti‐spike IgG with a distinct glycosylation pattern of their Fc tail at position 297, with low amounts of fucose and high amounts of galactose [8]. Decreased fucosylation of IgG antibodies is generally observed in response to surface‐exposed membrane‐embedded antigens [9], which has been described to occur in a variety of antiviral immune responses, such as in dengue virus [10] or HIV infected individuals [11]. However, a characteristic of severe COVID‐19 is the induction of a hyperinflammatory response by human alveolar macrophages through antibody‐dependent inflammation (ADI), leading to tissue damage, pulmonary edema, and coagulopathy [6, 7, 8, 12].

In recent years, it has become increasingly clear that changes in cellular metabolism are an essential mechanism actively shaping the immune response [13]. Proinflammatory macrophages are characterized by an increased dependency on glycolysis and pentose phosphate pathway (PPP), combined with a decreased utilization of the TCA cycle, β‐oxidation, and oxidative phosphorylation (OXPHOS) [14, 15, 16]. In contrast, macrophages with a wound‐healing phenotype mainly depend on an intact TCA cycle and OXPHOS as the main energy source [15, 16, 17].

Crosstalk of IC‐induced FcR signaling and Toll‐like receptor (TLR) activation plays a crucial role in boosting effector function of myeloid cells, such as proinflammatory cytokine induction in various tissues [18, 19]. For human dendritic cells, we previously identified that FcR‐mediated cytokine secretion strongly relies on IRF5‐regulated metabolic reprogramming toward a highly glycolytic phenotype [20]. Furthermore, rapid upregulation of glycolytic flux is an essential factor for fueling fatty acid synthesis and driving myeloid cell activation in a proinflammatory milieu [21].

Several studies have already revealed the impact of metabolic changes in SARS‐CoV‐2 infection and replication [22, 23, 24]. However, the role of metabolic reprogramming in pathogenic antibody‐mediated inflammation in the context of severe COVID‐19 is not yet well defined. In this study, we investigated metabolic reprogramming of human alveolar‐like macrophages upon exposure to pathological antibodies and the role of these metabolic changes in the development of hyperinflammation. We found that specifically anti‐spike IgG, but not viral stimuli, induced pronounced changes in cellular metabolism in human alveolar‐like macrophages. Moreover, we identified that inhibition of these metabolic pathways efficiently blocks anti‐spike IgG‐induced proinflammatory cytokine induction, and thus may be a promising therapeutic target to counteract the effects of pathogenic antibodies in patients experiencing severe COVID‐19.

## 2 Methods

**Key resources table**

**Table:** 

Reagent or resource	Source	Identifier
Antibodies		
COVA1‐18 WT	Brouwer et al. [25]	https://doi.org/10.1126/science.abc5902
Severe COVID‐19 patient serum	Amsterdam UMC COVID‐19 Biobank	N/A
Glut1 – Dylight 405	Heieis et al. [26]	https://doi.org/10.1038/s41467‐023‐41353‐z
PKM—PE	Heieis et al. [26]	https://doi.org/10.1038/s41467‐023‐41353‐z
SDHA—AF647	Heieis et al. [26]	https://doi.org/10.1038/s41467‐023‐41353‐z
CPT1A—PE‐Cy5	Heieis et al. [26]	https://doi.org/10.1038/s41467‐023‐41353‐z
ACC1 – Pe‐Cy7	Heieis et al. [26]	https://doi.org/10.1038/s41467‐023‐41353‐z
G6PD—APC‐Cy7	Heieis et al. [26]	https://doi.org/10.1038/s41467‐023‐41353‐z
ATP5a—Dylight 488	Heieis et al. [26]	https://doi.org/10.1038/s41467‐023‐41353‐z
CD36 – BV605	BD Biosciences	Cat# 563518
CD98 – BUV395	BD Biosciences	Cat# 744508
Human TruStain FcX	Biolegend	Cat# 422301
HIF‐1α	Invitrogen	Cat# 12‐7528‐80
FcγIIb block	Biolegend	Cat# 398302
Chemicals, peptides, and recombinant proteins		
Human M‐CSF	Miltenyi Biotec	Cat#130‐096‐491
Recombinant Human IL‐10 Protein	R&D Systems	Cat# 217‐IL‐025/CF
Recombinant SARS‐CoV2‐Spike Wuhan Hu‐1 Protein	Caniels et al. [27]	GenBank accession MN908947.3
Zombie NIR Fixable Viability Kit	Biolegend	Cat# 423106
2‐DG	MedChemExpress.com	Cat# HY‐13966
BPTES	MedChemExpress.com	Cat# HY‐12683
C75	MedChemExpress.com	Cat# HY‐12364
Etomoxir	MedChemExpress.com	Cat# HY‐50202
6‐AN	MedChemExpress.com	Cat# HY‐W010342
Oligomycin	Sigma‐Aldrich	Cat# 75351–5MG
UK‐5099	MedChemExpress.com	Cat# HY‐15475
Polyinosinic:polycytidylic acid	Sigma‐Aldrich	Cat#P1530
MitoTracker Deep Red FM	ThermoFisher	Cat#M224626
Critical commercial assays		
CD14 MicroBeads, human	Miltenyi Biotec	Cat#130‐050‐201
MitoProbe TMRM Assay Kit for Flow Cytometry	ThermoFisher	Cat#M20036
2‐(N‐(7‐Nitrobenz‐2‐oxa‐1,3‐diazol‐4‐yl)Amino)‐2‐Deoxyglucose) (2‐NBDG)	ThermoFisher	Cat# N13195
4,4‐Difluoro‐4‐bora‐3a,4a‐diaza‐s‐indacene (BIODIPY)	ThermoFisher	Cat# D3821
ELISA MAX Standard Set Human IL‐6	BioLegend	Cat#430501
Software and algorithms		
GraphPad Prism version 9.4.0	GraphPad Software	www.graphpad.com
SpetroFlo v.5	Cytek	www.cytekbio.com
FlowJo v. 10	BD Biosciences	www.flowjo.com

### 2.1 Monocyte‐Derived Alveolar Macrophage‐Like Macrophages

Buffy coats from healthy anonymous donors were purchased from Sanquin blood supply (Amsterdam, the Netherlands). All donors provided written consent prior to blood donation. Briefly, monocytes were isolated via Lymphoprep isolation (Stemcell) followed by magnetic bead separation using the MACS cell separation system (Miltenyi). The purified monocytes were differentiated for 6 days in Iscove's modified Dulbecco's medium (IMDM, Gibco) containing 5% fetal calf serum (CAPRICORN) and Penicillin/Streptomycin (Thermo Fisher) supplemented with 50 ng/mL human M‐CSF (Miltenyi). The culture medium was refreshed after three culture days. To generate an alveolar macrophage‐like phenotype, the medium was replaced by culture medium containing 50 ng/mL human IL‐10 (R&D) 24 h prior to stimulation.

### 2.2 Coating

Stabilized recombinant SARS‐CoV‐2 spike protein and monoclonal anti‐spike IgG1 (COVA1‐18) were generated as previously described [25, 27]. Spike protein‐specific ICs were generated by coating 2 µg/mL spike protein overnight on 96‐well high‐affinity plates (Nunc). Subsequently, the plates were blocked with 10% FCS in PBS for 1 h at 37°C to prevent unspecific binding. Monoclonal anti‐spike IgG1 (2 µg/mL) or severe COVID‐19 patient serum (1% in PBS) was added to the stimulation plates and incubated for 1 h at 37°C, followed by washing the plates with PBS to remove unbound protein.

### 2.3 Cell Stimulation and Inhibitor Treatment

All inhibitors were purchased in powdered form and dissolved according to the manufacturer's instructions. Human macrophages were diluted to a concentration of 277,777 cells/mL and pretreated with the indicated inhibitors (or DMSO as a control) for 30 min at 37°C. After preincubation, macrophages were added to the stimulation plate supplemented with either culture medium or with 20 µg/mL polyinosinic:polycytidylic acid (poly(I:C), Sigma‐Aldrich). For the FcγRIIb block, cells were preincubated with 20 µg/mL anti‐FcγRIIb (BioLegend Cat# 398302) for 30 min at 4°C. Prior to stimulation, this was adjusted to a final concentration of 5 µg/mL.

### 2.4 Enzyme‐Linked Immunosorbent Assay

To determine cytokine production, supernatants of the simulated human macrophages were harvested after 6 or 24 h. IL‐6 concentration was measured using antibody pairs from BioLegend (ELISA MAX Standard Set Human IL‐6, 430501).

### 2.5 Seahorse Metabolic Analysis

Spike protein‐specific ICs were generated in an XFe96 well Seahorse plate (Agilent) as described above. Prior to the experiment, the Seahorse XFe96 cartridge was hydrated with Seahorse XF calibrant (Agilent). A total of 50,000 alveolar‐like macrophages per well were added to the Seahorse plate in XF assay medium on RPMI basis (Sigma) supplemented with 2 mM glutamine (ThermoFisher), 5% FCS, and with or without 20 µg/mL poly(I:C). Injection compounds were diluted in Seahorse XF medium and added to the previously prepared cartridge and loaded into the machine for calibration. Mito Stress Test Injections (final concentrations): 10 mM Glucose (Sigma Aldrich), 1.5 µM Oligomycin A (Cayman), 3 µM FCCP (Sigma Aldrich), 1 µM Rotenone (Sigma Aldrich) and Antimycin A (Sigma Aldrich). Glycolysis stress test injections: 10 mM Glucose (Sigma Aldrich), 1.5 µM Oligomycin A (Cayman), and 10 mM 2‐DG (Medchemxpress.com).

### 2.6 MitoTracker DR and TMRM Staining

Cells were stimulated as described above. Fifteen minutes before the stimulation time ended, CCCP was added to the CCCP control conditions in a final concentration of 50 µM. Cells were harvested and washed with prewarmed IMDM without supplements. Samples were stained with 2.5 nM Mito Tracker Deep Red (DR) and 3 nM tetramethylrhodamine methyl ester (TMRM) for 15 min at 37°C protected from light. After staining, the cells were once washed with IMDM + 1% FCS and twice with cold PBS. Life cells were stained using Zombie Violet Dye (BioLegend). Cells were measured immediately after the staining procedure at BD LSR Fortessa (BD Biosciences). Analysis was performed with FlowJo v.10 (BD Biosciences)

### 2.7 Glucose and Fatty Acid Uptake

Alveolar‐like macrophages were stimulated as mentioned above, and cells were harvested after 1 h of stimulation time. Cells were stained with 2‐NBDG (ThermoFisher) diluted in prewarmed 37°C PBS in a concentration of 100 µM and BODIPY C16 (ThermoFisher) in a concentration of 20 nM. Live cells were stained using Zombie‐NIR viability dye (Biolegend). Samples were acquired on a Cytek Aurora 5 L spectral flow cytometer. Spectral unmixing was performed with SpectroFlow v.5, and further analysis was executed with FlowJo v.10 (BD Biosciences).

### 2.8 Spectral Flowcytometry

Antibodies for metabolic targets were generated as previously described [26]. A total of 600,000 cells per condition were prestained with viability dye (BioLegend) and Fc‐blocking solution (BioLegend) for 20 min on ice. Following fixation with Fixation buffer (BioLegend) for 15 min on ice, surface marker staining was performed in FACS buffer for 1 h on ice. Cells were permeabilized with 1× Perm buffer (ThermoFisher). Followed by intracellular metabolic targets staining and HIF‐1α (Invitrogen, Cat# 12‐7528‐80) staining for 2 h at RT. Samples were acquired on a Cytek Auora 5L spectral flow cytometer. Spectral unmixing was performed using SpetroFlow v.5. Samples were further analyzed with FlowJo v.10 (BD Biosciences).

### 2.9 Propidium Iodide (PI) Assay

Human macrophages were cultured in the presence of the selected metabolic inhibitors for 24 h. Following the inhibitor treatment, the supernatant was replaced with serum‐free IMDM supplemented with 3 µM PI reagent (Sigma‐Aldrich, P4170). After 30 min incubation under culture conditions, extracellular DNA content was measured by analyzing fluorescence intensities at *λ*_ex_/*λ*_em_ = 530/620 nm.

### 2.10 Lactate Assay

Supernatant of stimulated human macrophages was deproteinized by the addition of a final concentration of 3% w/v metaphosphoric acid (MPA). Followed by incubation with 27 mM NAD (Roche) solution in 0.5 M glycine‐0.4 M hydrazine buffer (pH 9.0). Lactate production was measured by lactate dehydrogenase (LDH, Roche) treatment. After 30 min incubation time with LDH at RT, lactate content was measured by NADH fluorescence at *λ*_ex_/*λ*_em_ 340/450.

### 2.11 Quantification and Statistical Analysis

Statistical significance of the data was determined using GraphPad Prism 9.4.0 (GraphPad). The statistical analysis applied to each figure is stated in the corresponding figure legend.

## 3 Results

### 3.1 Individual Stimulation with Anti‐Spike IgG Rapidly Boosts Glycolysis and Oxidative Phosphorylation

**FIGURE 1:**
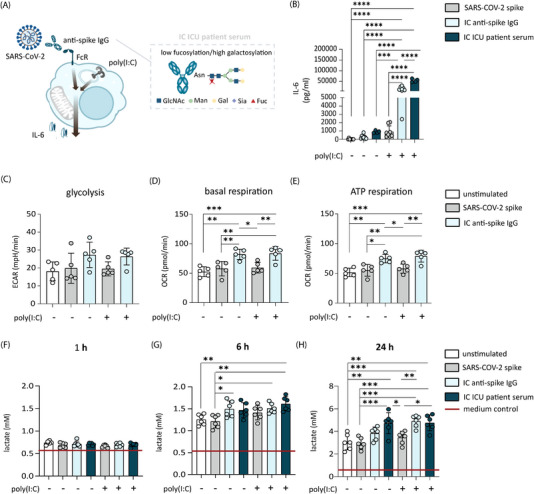
Stimulation with anti‐SARS‐CoV‐2 spike IgG rapidly boosts glycolysis and OXPHOS in human alveolar‐like macrophages. (A) anti‐spike IgG ICs induce proinflammatory cytokine induction of alveolar macrophages in combination with TLR activating signal (poly(I:C). Antibodies derived from COVID‐19 ICU patients are characterized by an aberrant glycosylation pattern. (B) IL‐6 production by human alveolar‐like macrophages after 24 h stimulation with ICs from recombinant anti‐spike IgG or serum from unvaccinated patients diagnosed with severe COVID‐19 (mean + SD). (C) Glycolysis, (D) basal respiration, and (E) ATP production of human alveolar‐like macrophages. Stimulation for 1 h followed by Seahorse assay (*n* = 5, mean + SD). (F–H) Lactate production of human alveolar‐like macrophages was stimulated as indicated for 1, 6, and 24 h (*n* = 6, mean + SD). IMDM medium without cells was added as control (red line). Significant differences were calculated with one‐way ANOVA. *p < 0.05; **p < 0.01; ***p < 0.001; ****p < 0.0001.

To assess if stimulation with anti‐spike IgG ICs changes core metabolic pathways in the context of severe COVID‐19, we examined changes in glycolysis and OXPHOS of monocyte‐derived alveolar‐like macrophages after short‐term exposure. Previous data have shown that in vitro stimulation of monocyte‐derived alveolar‐like human macrophages with anti‐spike IgG ICs serves as a promising experimental model to study antibody‐induced inflammation of alveolar macrophages in severe COVID‐19 [8]. We tested the effect of the recombinant anti‐spike IgG COVA1‐18, which we generated previously from B cells isolated from a patient with COVID‐19 [28]. In addition, we generated anti‐spike‐IgG ICs by incubating SARS‐CoV‐2 spike–coated wells with diluted serum from patients with severe COVID‐19 treated in the first pandemic wave at the intensive care unit (ICU) at the Amsterdam University Medical Center (UMC) [8]. In line with previous studies [8, 29], stimulation of alveolar‐like macrophages with only viral stimulus poly(I:C) (reflecting the first phase of infection), did not elevate proinflammatory cytokine production. In contrast, upon co‐stimulation of cells with a viral stimulus and anti‐spike IgG ICs, mimicking the postseroconversion phase of severe COVID‐19 infection, high amounts of proinflammatory cytokine IL‐6 were secreted (Figure 1A,B).

Extracellular metabolic flux analysis of human macrophages treated with anti‐spike IgG ICs showed an increased but not significant change in extracellular acidification rate (ECAR), indicative of a slightly enhanced glycolysis (Figure 1C; Figure S1A).

In proinflammatory macrophages, elevated glycolysis is associated with a decreased OXPHOS‐associated metabolism [15, 30]. Interestingly, the extracellular metabolic flux data revealed a rapid increase in oxygen consumption rate (OCR) representative of elevated OXPHOS utilization after anti‐spike IgG IC stimulation (Figure 1D,E; Figure S1B). Thus, our data indicate that anti‐spike IgG IC stimulation induces upregulation of both core metabolic pathways that boost ATP synthesis. Remarkably, in contrast to proinflammatory cytokine induction, which requires a strong interplay of FcR signaling and TLR‐activating signal, anti‐spike IgG ICs alone were sufficient to induce changes in glycolysis and basal respiration (Figure 1C–E; Figure S1 A,B), and poly(I:C) had no synergistic effect.

Macrophages resembling a proinflammatory phenotype are classically characterized by an increased dependence on glycolysis, fatty acid synthesis, and the PPP [15, 17]. In this proinflammatory condition, pyruvate is reduced into lactate instead of being used to fuel the TCA cycle [15, 31]. Thus, lactate secretion can serve as a simple measure to determine alterations of the glycolytic pathway and reduced pyruvate metabolism in activated macrophages [32]. To further investigate the kinetics of anti‐spike IgG ICs‐induced metabolic reprogramming, we measured lactate secretion in the medium of anti‐spike IgG‐stimulated macrophages after 1, 6, and 24 h activation time. After 1 h of stimulation time, no differences between the stimulation conditions could be observed (Figure 1F). In the next time frame, after 6 h of stimulation time, a small increase in lactate production in the samples stimulated with recombinant anti‐spike IgG ICs or ICs derived from ICU patient serum could be detected (Figure 1G). Twenty‐four hours after activation, the secretion of lactate in the anti‐spike IgG ICs‐treated cells was further significantly elevated (Figure 1H). In line with the extracellular metabolic flux data, these changes were mainly induced by anti‐spike IgG stimulation, as the addition of a co‐stimulatory TLR‐activating agent did not further affect lactate secretion.

Since antibody stimulation significantly increased glycolytic activity and OXPHOS of alveolar‐like macrophages, we assessed whether glucose and fatty acid uptake would be increased upon stimulation as well. We visualized glucose and fatty acid uptake by tracking the uptake of fluorescent glucose 2‐NBDG and fluorescent BODIPY, respectively. There was a trend toward increased 2‐NBDG uptake after 1 h of stimulation in the conditions stimulated with anti‐spike IgG immune complexes (Figure S1C); however, these changes did not reach statistical significance. Furthermore, no changes in fatty acid uptake were observed between the different stimulation conditions (Figure S1D).

An important driver of glycolytic metabolism and release of proinflammatory cytokines is hypoxia‐induced factor (HIF)‐1α [33, 34]. Therefore, we investigated whether anti‐spike IgG IC stimulation changed HIF‐1α expression in human alveolar‐like macrophages. After 1 h of stimulation time, no differences in HIF‐1α protein expression were observed between the different conditions (Figure S1E). In contrast, after 24 h of stimulation time, there was a trend toward elevated HIF‐1α expression in anti‐spike IgG IC‐treated conditions (Figure S1F). Interestingly, as previously observed in the metabolic flux data, individual anti‐spike IgG IC treatment was sufficient to induce this effect, and no further increase was observed in cells additionally treated with co‐stimulatory signal poly(I:C).

Taken together, these data indicate that anti‐spike IgG IC stimulation induces upregulation of both metabolic core pathways, boosting ATP synthesis, that is, glycolysis and OXPHOS.

### 3.2 Anti‐Spike IgG Induces Rapid Upregulation of Specific Metabolic Enzymes

**FIGURE 2:**
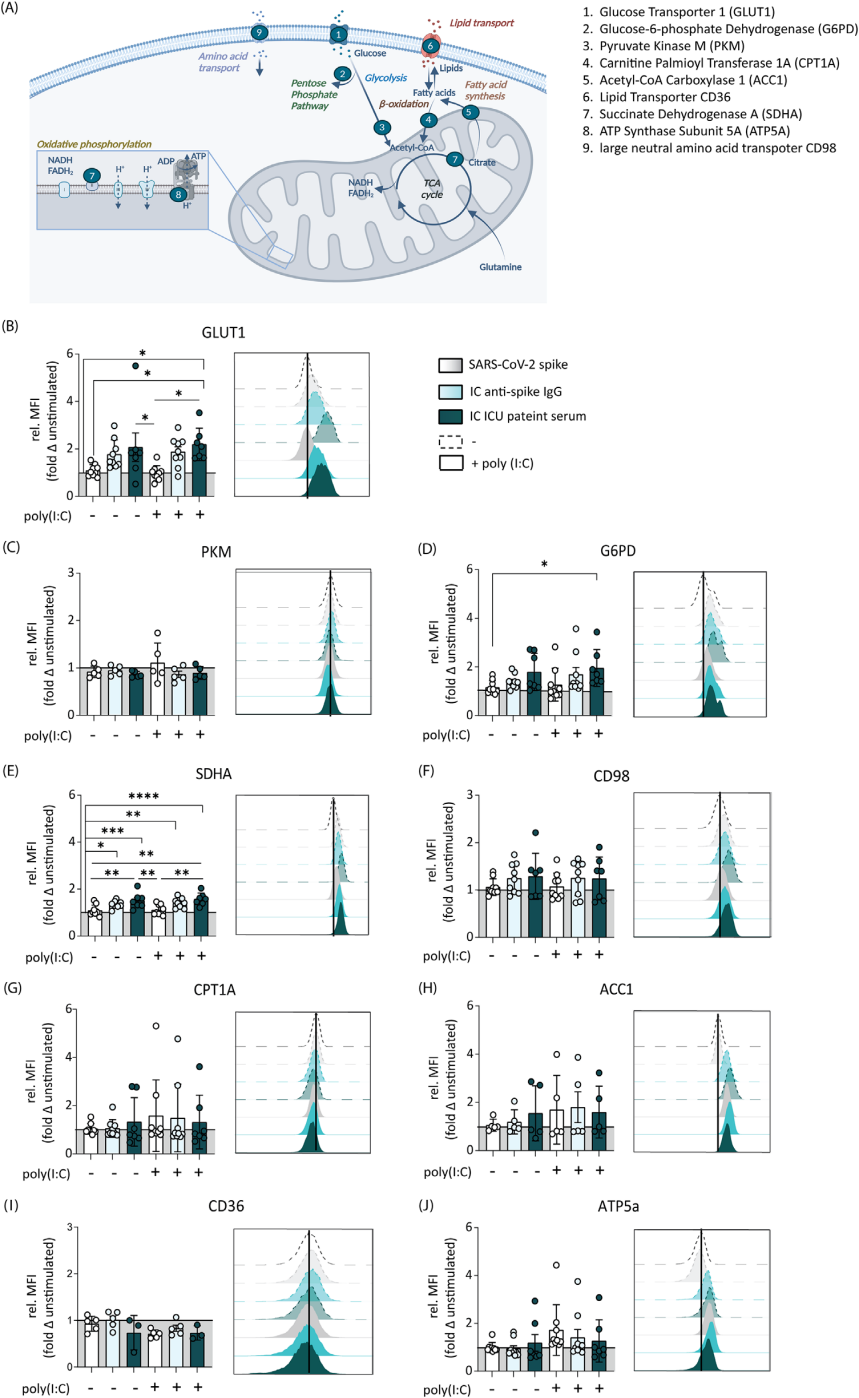
Anti‐spike IgG induces upregulation of specific metabolic enzymes. (A) Schematic representation of Metflow analysis targets. Created in BioRender. Geyer, C. (2024) BioRender.com/h10r049. (B–J) Changes in relative MFI of metabolic enzymes of human macrophages after 6 h stimulation with ICs of recombinant IgG or ICU patient serum with or without viral stimulus poly(I:C). Representative histograms (unstimulated indicated as black line) and data points from individual donors (*n* = 5–8, mean + SD). The gating strategy is shown in Figure S3. Significant differences were calculated with one‐way ANOVA. *p < 0.05; **p < 0.01; ***p < 0.001; ****p < 0.0001.

To further characterize changes induced in relevant metabolic pathways upon anti‐spike IgG IC stimulation, we analyzed the expression of a selection of core metabolic enzymes via Metflow analysis [26]. Briefly, human alveolar‐like macrophages were stimulated with anti‐spike IgG ICs as shown above (Figure 1A,B). Following 6 h stimulation, metabolic targets were stained and measured using spectral flow cytometry (Figure 2A). In line with the extracellular metabolic flux (Figure 1) and 2‐NBDG data (Figure S1D), stimulation of human macrophages with anti‐spike IgG ICs significantly increased the expression of glucose transporter GLUT1 (Figure 2B). Interestingly, even though stimulation with anti‐spike IgG increased GLUT1 expression and elevated the glycolytic flux, PKM expression was not significantly elevated (Figure 2C), potentially indicating an increased flux of glucose into upstream offshoots of glycolysis such as the PPP. Indeed, stimulation with anti‐spike IgG ICs induced elevated expression of PPP‐associated enzyme G6PD (Figure 2D). Furthermore, consistent with increased OXPHOS, anti‐spike IgG ICs promoted expression of SDHA (Figure 2E), an enzyme in the TCA cycle and complex II enzyme of the respiratory chain. Finally, a trend toward increased expression of amino acid transporter CD98 (Figure 2F) was visible after anti‐spike IgG IC stimulation, which became more prominent after 24 h stimulation time (Figure S2). No significant changes in the expression of metabolic enzymes CPT1A, ACC1, CD36, and ATP5a could be detected (Figure 2G–J). In line with the extracellular metabolic flux data and lactate assay, the changes in expression of G6PD, CD98, and SDHA were mainly induced by anti‐spike IgG IC stimulation and remained elevated for at least 24 h after stimulation (Figure S2).

Since Fc‐mediated antibody effector functions correlate with expression and type of FcR, we measured expression levels of the different FcγR on human alveolar‐like macrophages. As shown in Figure S4A, FcγRI (CD64) and FcγIII (CD64) were expressed at similar levels, while FcγRIIb/c (CD32 b/c) showed a slightly lower expression, and FcγRIIa/b (CD32a/b) showed increased expression on human alveolar‐like macrophages (Figure S4A). In contrast to other receptors of the FcR family expressed on human alveolar‐like macrophages, FcγRIIb (CD32b) cross‐linking inhibits activating signals by phosphorylation of its immunoreceptor tyrosine‐based inhibitory motif (ITIM) in the cytoplasmic domain [35]. We therefore determined whether a block of FcγRIIb would further amplify the metabolic changes induced by anti‐spike IgG IC. Notably, FcγRIIb inhibition indeed further increased GLUT‐1 expression of alveolar‐like macrophages, especially when additionally treated with costimulatory signal poly(I:C) (Figure S4B).

### 3.3 Anti‐Spike IgG Triggers Rapid Changes in Macrophage Mitochondria

**FIGURE 3:**
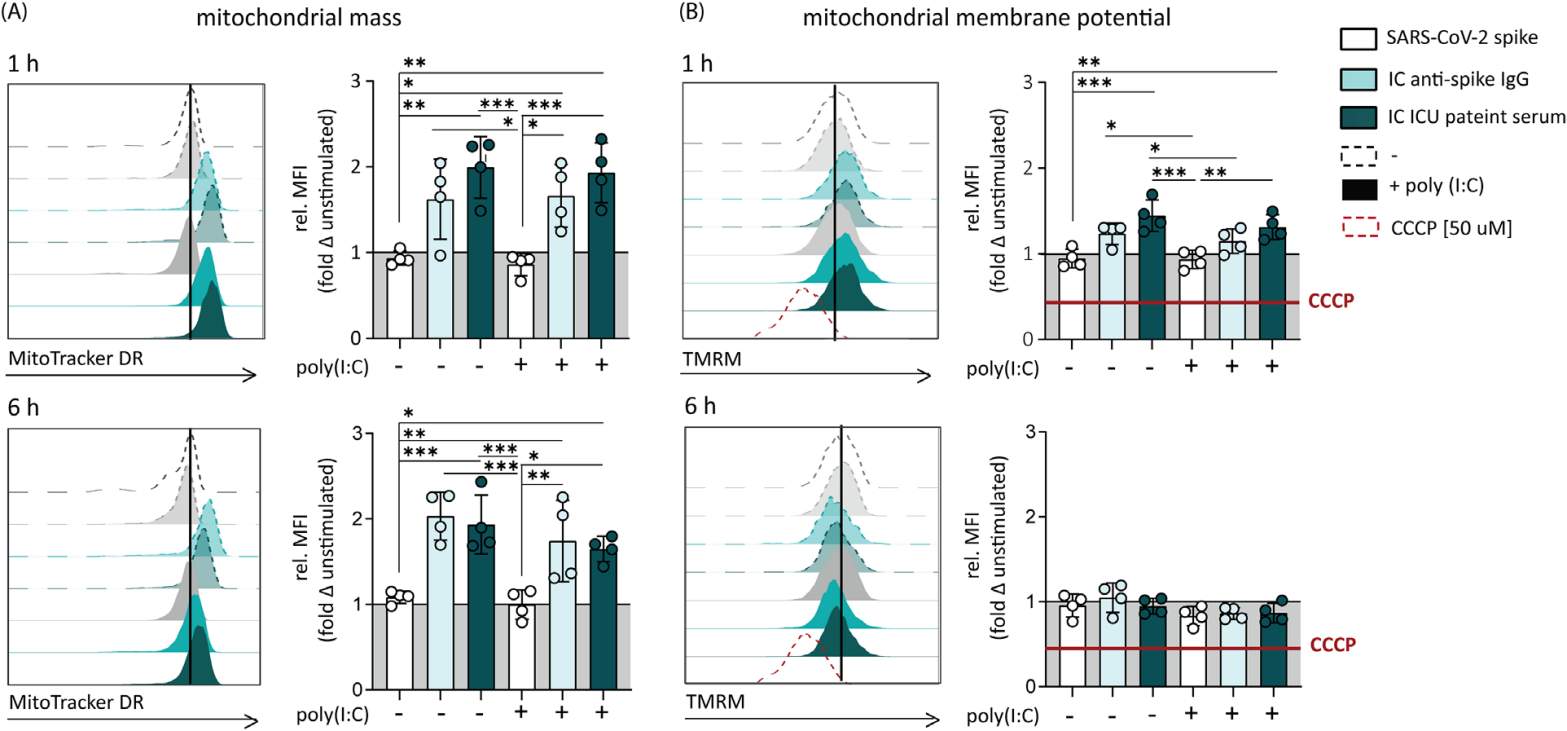
Anti‐spike IgG IC induces rapid changes in human macrophage mitochondria. Representative data of independent experiments (left, unstimulated indicated as black line) MitoTracker DR (A), TMRM (B). Data points from individual donors (right, *n* = 4, mean + SD). Human alveolar‐like macrophages were stimulated as indicated and immediately stained with MitroTracker DR (3 nM) and TMRM (3 nM). Fifteen minutes before stimulation end, 50 µM CCCP was added to induce mitochondrial depolarization in TMRM samples as negative control. Gating strategy is shown in Figure S3. Significant differences were calculated with one‐way ANOVA. *p < 0.05; **p < 0.01; ***p < 0.001; ****p < 0.0001.

To further understand the effect of pathogenic anti‐spike IgG on mitochondrial function of human alveolar‐like macrophages in severe COVID‐19, we analyzed mitochondrial mass and membrane potential in response to anti‐spike IgG IC stimulation, using Mito Tracker DR [36, 37] and TMRM probes [38], respectively. After 1 h of stimulation, human alveolar‐like macrophages stimulated with recombinant anti‐spike IgG and ICs derived from severe COVID‐19 ICU patient serum showed a significantly increased Mito Tracker DR signal (Figure 3A), as well as an elevated mitochondrial membrane potential (Figure 3B). After 6 h of stimulation time, the changes in Mito Tracker DR accumulation remained stable while the TMRM signal was decreased (Figure 3A,B).

Taken together, these data suggest that anti‐spike IgG stimulation induces rapid changes in mitochondrial membrane potential and mass, suggesting the first signs of mitochondrial damage, such as mitochondrial membrane depolarization.

### 3.4 Anti‐Spike IgG‐Induced Hyperinflammation Is Functionally Dependent on Glycolysis, PPP, and Fatty Acid Synthesis

**FIGURE 4:**
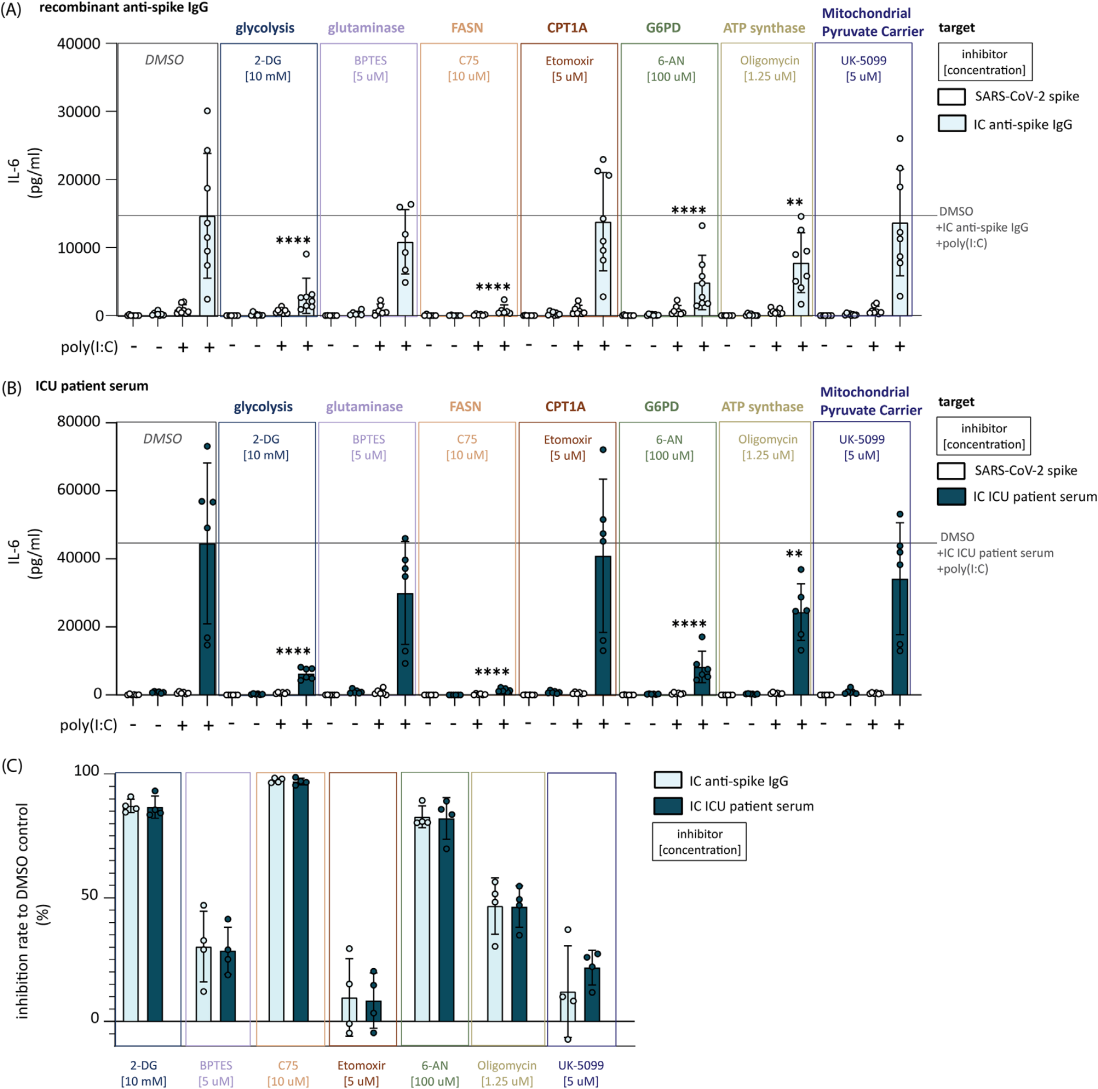
Anti‐spike IgG‐induced proinflammatory cytokine induction is functionally dependent on glycolysis, fatty acid synthesis, and PPP. (A) IL‐6 secretion of human alveolar‐like macrophages stimulated with anti‐spike IgG IC and poly(I:C) after treatment with the indicated metabolic inhibitors. Each data point represents one individual donor (*n* = 4–8, mean + SD). Stimulation with immune complexes derived from recombinant anti‐spike IgG (A) or ICU patient serum (B) inhibition rate compared with DMSO control (C). Significant differences were calculated with one‐way ANOVA. *p < 0.05; **p < 0.01; ***p < 0.001; ****p < 0.0001. Statistics indicate changes between inhibitor‐treated stimulation conditions compared with identical stimulation conditions in the DMSO control sample.

To assess if the identified changes in metabolic pathway activity are functionally required for anti‐spike IgG IC‐induced hyperinflammation, we tested the effect of metabolic pathway inhibitors on the proinflammatory cytokine induction of human alveolar‐like macrophages stimulated with anti‐spike IgG ICs with or without poly(I:C). In line with the extracellular metabolic flux and metabolic enzyme expression data (Figures 1 and 2), IL‐6 production induced by co‐stimulation of human alveolar‐like macrophages was blocked by inhibition of glycolysis (by 2‐DG) and PPP (by 6‐AN) (Figure 4A,B).

Interestingly, even though most donors did not show changes in ACC1 expression (Figure 2H), blocking fatty acid synthesis with C75 efficiently reduced IL‐6 production to the level of unstimulated macrophages, suggesting an important functional role of fatty acid synthesis in anti‐spike IgG IC‐induced inflammation. On the other hand, neither inhibition of the mitochondrial pyruvate carrier by UK‐5099 nor β‐oxidation using Etomoxir affected the antibody‐induced proinflammatory cytokine production (Figure 4A,B). Despite the strong upregulation of basal respiration seen in the extracellular flux assay (Figure 1D,E), blocking OXPHOS with oligomycin only induced a small but still significant decrease in IL‐6 induction (Figure 4). This may be explained by the kinetics of the anti‐spike IgG IC‐induced metabolic reprogramming, which was dependent on OXPHOS mainly early after stimulation (Figure 1D,E). Of note, none of the inhibitors induced cell death at the indicated concentrations (Figure S5).

Finally, we set out to validate these findings with anti‐spike IgG from severely ill patients by stimulating cells with serum obtained from first‐wave COVID‐19 ICU patients. Importantly, ICU patient serum displayed a similar dependency on glycolysis, fatty acid synthesis, and PPP to promote elevated IL‐6 induction (Figure 4B). Moreover, when we compared the percentage of inhibition, the metabolic inhibitors suppressed cytokine induction by recombinant and patient‐derived anti‐spike IgG in a strikingly similar manner (Figure 4C). This suggests that the higher cytokine induction by patient‐derived IgG (Figure 1B) results from quantitative, but not qualitative differences in metabolic reprogramming.

## 4 Discussion

Severe COVID‐19 is an immunological disorder, characterized by hyperinflammation of macrophages and monocytes that cause tissue destruction [8, 39], edema, and coagulopathy [12, 40]. While metabolic reprogramming of immune cells is critical for immune activation [13], the metabolic pathways that enable hyperinflammation in the context of severe COVID‐19 are still largely unknown. In this study, we set out to investigate the role of antibody‐induced metabolic reprogramming in severe disease progression to identify potential therapeutic targets for the treatment of severe COVID‐19.

In general, metabolic reprogramming toward high glycolysis provides important building blocks for transcription and promotes the generation of reactive oxygen species, which are beneficial for the host immune system, leading to a rapid and effective pathogen clearance [15, 41]. In contrast, in severe COVID‐19 patients, alteration of glucose metabolism, such as increased glycolysis in BALF macrophages [42, 43], is associated with pathogenic overactivation of the immune response and correlated with a poor disease prognosis [43, 44, 45, 46].

Here, we show that anti‐spike IgG ICs, as present upon seroconversion, may contribute to severe disease outcome by rapidly triggering metabolic reprogramming of alveolar macrophages toward a highly glycolytic phenotype. Blocking this glycolytic reprogramming by 2‐DG treatment counteracted the hyperinflammatory immune reaction. Thus, 2‐DG may be an efficient target to control the pathogenic overactivation in severe COVID‐19. In phases II and III clinical trials, performed in India in patients with moderate‐to‐severe COVID‐19 progression, treatment with 2‐DG showed alleviation of symptoms [47, 48]. These studies thus provide the first proof of principle that reducing glycolytic activity in severe COVID‐19 patients could have therapeutic potential. However, previous studies done in cancer patients have shown that 2‐DG can induce potential side effects, including hypoglycemia, nausea, and fatigue. However, these side effects were non‐life‐threatening if the doses were limited to a maximum of 63 mg/kg [49, 50]. Nevertheless, it is yet unclear whether 2‐DG treatment would interfere with the standard treatment protocol of severe COVID‐19, including antiviral drugs and systemic corticosteroids [51]. Therefore, while 2‐DG is a potentially promising treatment for severe COVID‐19, further evaluation of the optimal treatment doses, administration time, and target patient group needs to be performed in additional randomized clinical trials.

Our experiments also show that anti‐spike IgG IC strongly upregulates PPP‐associated enzyme G6PD in human macrophages. In addition, our data demonstrate that antibody‐induced cytokine secretion crucially depends on PPP activity, as inhibition of this pathway via 6‐AN treatment efficiently blocks anti‐spike IgG IC‐induced IL‐6. Polidatin, a molecule naturally occurring in *Polygonum cuspidatum*, directly inhibits G6PD [52]. Phase II clinical studies testing supportive treatment with Polydatin in the context of inflammatory bowel disease and endometriosis‐related pain indicated no toxic effects in humans for a treatment dose of 20–40 mg [53, 54]. Given the importance of the PPP for the generation of biosynthesis precursor molecules and NADPH, this pathway may be an additional promising therapeutic target to counterbalance the excessive cytokine induction in severe COVID‐19 patients [23]. Even though the PPP inhibitor Polidatin is a promising drug candidate due to its low toxicity in humans, further in vitro studies followed by randomized clinical trials would be required to evaluate efficiency and specificity in severe COVID‐19 disease treatment.

It has been established that inflammatory macrophages tend to rely on de novo fatty acid synthesis to maintain cellular functions, instead of increasing fatty acid uptake [55, 56, 57, 58, 59, 60]. Indeed, several studies have identified fatty acid synthesis as an essential link of metabolic reprogramming and cytokine secretion in myeloid cells [21, 57, 61, 62]. By fostering ER and Golgi expansion, fatty acid synthesis supports cytokine induction of myeloid cells in a proinflammatory milieu [21, 63]. Inhibition via C75 furthermore blocks IL‐1β secretion in murine macrophages in the context of sepsis [62]. This is further supported by recent studies where both ACC and fatty acid synthase (FASN) have been implicated in the metabolic rewiring of macrophages and in the subsequent synthesis of proinflammatory cytokines, such as IL‐6 and IL‐1β [64, 65]. In line with this, our data reveal no role for the process of lipid uptake in the inflammatory response (as evidenced by the lack of differences in BODIPY C16 staining), and instead show that inhibition of FASN via C75 effectively blocks anti‐spike IgG IC‐induced IL‐6 cytokine secretion, suggesting an important role for de novo fatty acid synthesis in antibody‐induced proinflammatory cytokine induction. Combined with the increased glycolysis and PPP enhancement, these data suggest a similar molecular mechanism underlying cytokine secretion as described for dendritic cells [21], thereby linking increased glycolytic flux and fatty acid synthesis as essential key pathways for proinflammatory cytokine production. Enhanced fatty acid synthesis was shown to induce NLRP3 inflammasome activation and proinflammatory cytokine synthesis [65]. Additionally, it has been suggested that fatty acid synthesis may also trigger increased membrane synthesis, supporting the ER and Golgi expansion required for the FcR‐triggered increase of pro‐inflammatory cytokine transcription [8, 20, 21]. The increased de novo fatty acid synthesis is fueled by NADPH produced by the elevated glycolytic flux, enhancing PPP activation [21]. Interestingly, in our dataset, ACC1 expression showed a mixed pattern of increased expression in some donors upon anti‐spike IgG IC stimulation, while in other donors, the expression levels remained stable. ACC1 expression is reported to mainly reflect the cell's ability for fatty acid synthesis [66]. Since obese severely ill COVID‐19 patients show aberrant levels of blood lipids [67] and altered ACC1 levels have been associated with the development of obesity [68], the role of ACC1 expression in the development of severe COVID‐19 disease progression may be of interest for further studies.

High levels of the proinflammatory cytokine IL‐6 are a key factor in the progression of severe COVID‐19 and are associated with increased mortality [69, 70]. Even though antibody‐induced proinflammatory cytokine induction requires a strong cross‐talk between FcR and TLR signaling [8], our data indicate that anti‐spike IgG IC‐mediated metabolic reprogramming is mainly controlled by FcR signaling. This is in line with a recent study that demonstrated that FcR‐mediated increase in glycolysis boosts inflammation in lupus nephritis [71]. This FcR‐mediated glycolytic switch, independent of a costimulatory signal, may therefore be relevant for inflammatory responses beyond the context of COVID‐19, including rheumatoid arthritis (RA) [72], Sjögren's Syndrome [73], or systemic lupus erythematosus (SLE) [71].

IgG affinity toward FcRs and affiliated induction of effector functions are strongly dependent on changes in the antibody glycosylation pattern [74, 75]. Afucosylated antibodies from patients amplify inflammation by increased binding to FcγRIII [76], while recombinant anti‐spike IgG (with conventional Fc glycosylation) predominantly activates FcγRIIa signaling [8]. Interestingly, in contrast to cytokine secretion, our data indicate that metabolic reprogramming induced by anti‐spike IgG is not influenced by alterations in antibody glycosylation pattern (Figure 4). This may relate to overlapping signaling pathways downstream of FcRs. Both FcγRIIa and FcγRIII activate Syk and PI3K signaling upon antibody crosslinking [77, 78], which have been identified as key molecules that mediate anti‐spike IgG IC‐induced hyperinflammatory responses by alveolar macrophages [8, 29]. Data by Jing et al. [71] indicate that FcR‐mediated glycolytic activation is functionally dependent on Syk and PI3K activation, suggesting a potential molecular link between FcγR activation and metabolic reprogramming via Syk and PI3K activation. Interestingly, as opposed to the activating FcγRs that promote metabolic rewiring, we find the inhibitory receptor FcyRIIb does the opposite, suggesting that the net degree and possible nature of FγcR‐driven metabolic alterations are shaped by the balance between engagement of activating and inhibitory FcγRs.

Mitochondrial dysfunction plays a role in increased stress and inflammation, contributing to the worsening of symptom severity in COVID‐19 patients [79, 80, 81, 82]. In addition, risk groups such as elderly patients are characterized by generally reduced mitochondrial health [83]. Our data show that rapidly in the kinetics of the anti‐spike IgG‐induced inflammatory response, alveolar‐like macrophage mitochondria show first signs of altered membrane potential and function. After 1 h of stimulation, the TMRM signal and respiration in anti‐spike IgG IC‐stimulated cells were increased, indicating that the initial response is characterized by increased mitochondrial activity. However, this appeared not to be functionally relevant for heightened cytokine production, as oligomycin treatment has minimal effects on this. Interestingly, after 6 h of stimulation, the first signs of loss of TMRM staining were visible, indicating the presence of depolarized mitochondria, and an indication of a loss of mitochondrial activity and potentially damage [84]. Of note, the Mito Tracker staining was increased at time points 1 and 6 h upon stimulation with anti‐spike IgG ICs. Given that mitochondrial biosynthesis in macrophages is reported to occur in a time frame of several hours [85], these changes are probably not induced by increased mitochondrial biosynthesis but may rather reflect changes in mitochondrial morphology. Since mitochondrial damage has been associated with symptoms persisting even after the virus has been cleared [86, 87, 88], further investigation of the role of anti‐spike IgG ICs in the development of mitochondrial dysfunction may be of interest for further studies, even beyond macrophages. For instance, platelets of severe COVID‐19 patients have also been reported to display signs of damaged mitochondria and bioenergetics failure [89]. Given that platelets express FcγRIIa and pathogenic antibodies of severe COVID‐19 patients can directly activate platelets [90] to contribute to thrombosis in severe COVID‐19 [12], it is conceivable that metabolic reprogramming by IgG IC during severe COVID‐19 applies to a wider range of cells.

A limitation of our study is that the in vitro conditions of our model are not able to completely mimic the metabolic niche of alveolar macrophages in severe COVID‐19 patients. Furthermore, since it was not possible to analyze bronchoalveolar lavage (BAL) macrophages derived from severe COVID‐19 patients, we used a model closely mimicking alveolar macrophages based on transcriptomic signature [91] as well as on alveolar macrophage surface marker expression (data not shown). However, the use of healthy donors for the generation of alveolar‐like macrophages does not take into account potential pre‐existing metabolic deficiencies in alveolar macrophages of severe‐COVID‐19‐risk‐group individuals.

Taken together, we here show that the hyperinflammatory response induced in the context of severe COVID‐19 critically depends on anti‐spike IgG IC‐induced metabolic reprogramming of macrophages toward a highly glycolytic phenotype. This mechanism is mainly dependent on FcγR signaling and characterized by rapid kinetics. Moreover, we show that IgG IC‐mediated inflammation can be counteracted by inhibiting glycolysis, fatty acid synthesis, and the pentose phosphate pathway. Therefore, these metabolic pathways may be promising targets to counteract the effects of pathological antibodies in patients suffering from severe COVID‐19.

## Author Contributions

Conceptualization: Riekelt H. Houtkooper, Bart Everts, and Jeroen den Dunnen; Methodology: Chiara E. Geyer, Luís Almeida, Graham A. Heieis, Riekelt H. Houtkooper, Bart Everts, Jeroen den Dunnen; Validation, formal analysis, and writing – original draft: Chiara E. Geyer; Investigation: Chiara E. Geyer, Luís Almeida, Lynn Mes, Frank Otto, W. Ashwin Mak, and Hung‐Jen Chen; Resources: Luís Almeida, Frank Otto, W. Ashwin Mak, Graham A. Heieis, Jennifer Veth, Steven W. de Taeye, Tom G. Caniels, Tom P. L. Bij, Marit J. van Gils, Menno de Winther, Amsterdam UMC COVID‐19 Biobank, Jan Van den Bossche; Writing – Review and Editing: Luís Almeida, Lynn Mes, Frank Otto, W. Ashwin Mak, Graham A. Heieis, Steven W. de Taeye, Tom G. Caniels, Tom P. L. Bij, Marit J. van Gils, Menno de Winther, Jan Van den Bossche, Riekelt H. Houtkooper, Hung‐Jen Chen, Bart Everts, Jeroen den Dunnen; Visualization: Chiara E. Geyer; Supervision: Riekelt H. Houtkooper, Bart Everts, Jeroen den Dunnen; Funding acquisition: Bart Everts, Jeroen den Dunnen.

## Funding

JdD was supported by ZonMW (10430 01 201 0008), Amsterdam Infection and Immunity COVID‐19 grant (24184), AMC Fellowship (2015), European Union's Horizon 2020 research and innovation programme (847551), AGEM matching grant (2020), and Innovative Medicines Initiative 2 Joint Undertaking grant (831434). BE was supported by funding from the European Union's Horizon 2020 research and innovation program under the Marie Skłodowska‐Curie Grant agreement no. 812890. The funders had no role in the design, data collection, data analysis, and reporting of this study.

## Ethics Statement

This study protocol was reviewed and approved by Commissie Toetsing Biobanken (CTB, Amsterdam UMC, The Netherlands), approval number 2023.0978. All participants provided written informed consent to Amsterdam UMC COVID‐19 Biobank. Buffy coats from healthy donors were purchased from Sanquin blood supply (Amsterdam, the Netherlands). Donors provided written informed consent prior to blood donation to Sanquin.

## Conflicts of Interest

The authors declare no conflicts of interest.

## Supporting information




**Supporting File 1**: eji70087‐sup‐0001‐SuppMat.pdf.

## Data Availability

All data associated with this study are presented in the paper or supplementary materials. The recombinant anti‐Spike IgG1 antibody COVA1‐18 is available upon request to the corresponding authors through a materials transfer agreement. Further information and requests for resources and reagents should be directed to and will be fulfilled by the corresponding author, Jeroen den Dunnen (j.dendunnen@amsterdamumc.nl).
