# Supplementary material for: Hyperinflammation by Human Macrophages Induced by SARS‐CoV‐2 Anti‐Spike IgG Is Dependent on Glucose and Fatty Acid Metabolism
Source: Eur J Immunol. 2025 Dec 19;55(12):e70087. doi: 10.1002/eji.70087 (PMC12716191; doi:10.1002/eji.70087)
Supplement: Supplementary file 1 — Supporting File 1: eji70087‐sup‐0001‐SuppMat.pdf. [file EJI-55-e70087-s001.pdf]

Supplementary

Table S1. Members and affiliation of the Amsterdam UMC COVID-19 Biobank

Name	Affiliations
M.A. van Agtmael	Department of Infectious Diseases
A.G. Algera	Department of Intensive Care
B. Appelman	Department of Infectious Diseases
F.E.H.P. van Baarle	Department of Intensive Care
D. van de Beek	Department of Neurology
M. Beudel	Department of Neurology
H J Bogaard	Department of Pulmonology
M. Bomers	Department of Infectious Diseases
P.I. Bonta	Department of Pulmonology
L.D.J. Bos	Department of Intensive Care
M. Botta	Department of Intensive Care
J. de Brabander	Department of Infectious Diseases
G.J. de Bree	Department of Infectious Diseases
M.C. Brouwer	Department of Neurology
S. de Bruin	Department of Intensive Care
M. Bugiani	Department of Pathology
E.B. Bulle	Department of Intensive Care
O. Chouchane	Department of Infectious Diseases
A.P.M. Cloherty	Experimental Immunology
D. Buis	Department of Infectious Diseases
M. C.F.J. de Rotte	Department of Clinical Chemistry
M. Dijkstra	Department of Clinical Chemistry
D.A. Dongelmans	Department of Intensive Care
R.W.G Dujardin	Department of Intensive Care
P.E. Elbers	Department of Intensive Care
L.M. Fleuren	Department of Intensive Care
S.E. Geerlings	Department of Infectious Diseases
T.B.H. Geijtenbeek	Department of Experimental Immunology
A.R.J. Girbes	Department of intensive care
A. Goorhuis	Department of Infectious Diseases
M.P. Grobusch	Department of Infectious Diseases
L.A. Hagens	Department of Intensive Care
J. Hamann	Amsterdam UMC Biobank Core Facility
V. C. Harris	Department of Infectious Diseases
R. Hemke	Department of Radiology
S.M. Hermans	Department of Infectious Diseases
L.M.A. Heunks	Department of Intensive Care
M.W. Hollmann	Department of Anesthesiology
J. Horn	Department of Intensive Care
J.W. Hovius	Department of Infectious Diseases
M.D. de Jong	Department of Medical Microbiology
R. Koing	Department of Neurology
E.H.T. Lim	Department of Intensive Care
N. van Mourik	Department of Intensive Care
J.F Nellen	Department of Infectious Diseases
E.J. Nossent	Department of Pulmonology
F. Paulus	Department of Intensive Care
E. Peters	Department of Infectious Diseases
D. Piña-Fuentes	Department of neurology
T. van der Poll	Department of Infectious Diseases
B. Preckel	Department of Anesthesiology
S.J. Raasveld	Department of Intensive Care
T.D.Y. Reijnders	Department of Infectious Diseases
M. Schinkel	Department of Infectious Diseases
F.A.P. Schrauwen	Department of Clinical Chemistry

M.J. Schultz	Department of Intensive Care
A.R. Schuurman	Department of Internal Medicine
J. Schuurmans	Department of Intensive Care
K. Sigaloff	Department of Infectious Diseases
M.A. Slim	Department of Intensive Care and Infectious Diseases
P. Smeele	Department of Pulmonology
M.R. Smit	Department of Intensive Care
C. Stijnis	Department of Infectious Diseases
W. Stilma	Department of Intensive Care
C.E. Teunissen	Neurochemical Laboratory
P. Thoral	Department of Intensive Care
A.M. Tsonas	Department of Intensive Care
P.R. Tuinman	Department of Intensive Care
M. van der Valk	Department of Infectious Diseases
D.P. Veelo	Department of Anesthesiology
A.P.J. Vlaar	Deparment of Intensive Care
C. Volleman	Department of Intensive Care
H. de Vries	Department of Intensive Care
L.A. van Vught	Department of Intensive Care and Infectious Diseases
M. van Vugt	Department of Infectious Diseases
W.J. Wiersinga	Department of Infectious Diseases
D. Wouters	Department of Clinical Chemistry
A.H. Zwinderman	Department of Clinical Epidemiology, Biostatistics and Bioinformatics

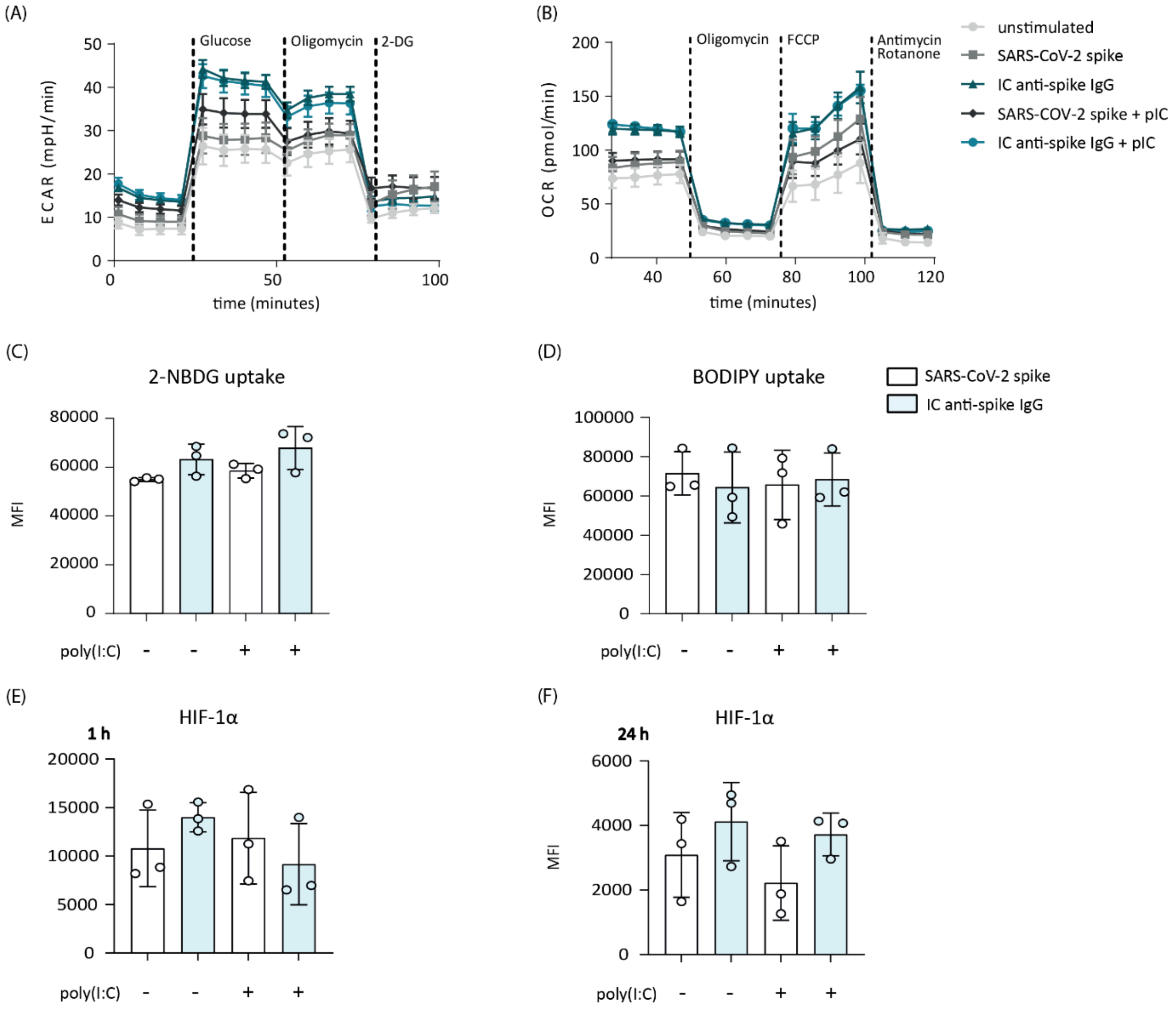

Figure S1: Metabolic changes of alveolar-like macrophages induced by anti-spike IgG stimulation (A) Real-time changes in ECAR (A) and OCR (B) of human alveolar-like macrophages stimulated with ICs from recombinant anti-spike IgG as indicated for a stimulation time of 1 h (Representative donor, mean + SEM). (C) glucose uptake quantified by glucose analogue 2-NBDG uptake (C), and fatty acid uptake, quantified by BIODIPY uptake (D) (n=3, mean + SD) of human alveolar-like macrophages after 1 h of stimulation time. (E) changes of HIF-1α expression in alveolar-like macrophages upon stimulation after 1 h and 24 h (n=3, mean + SD).

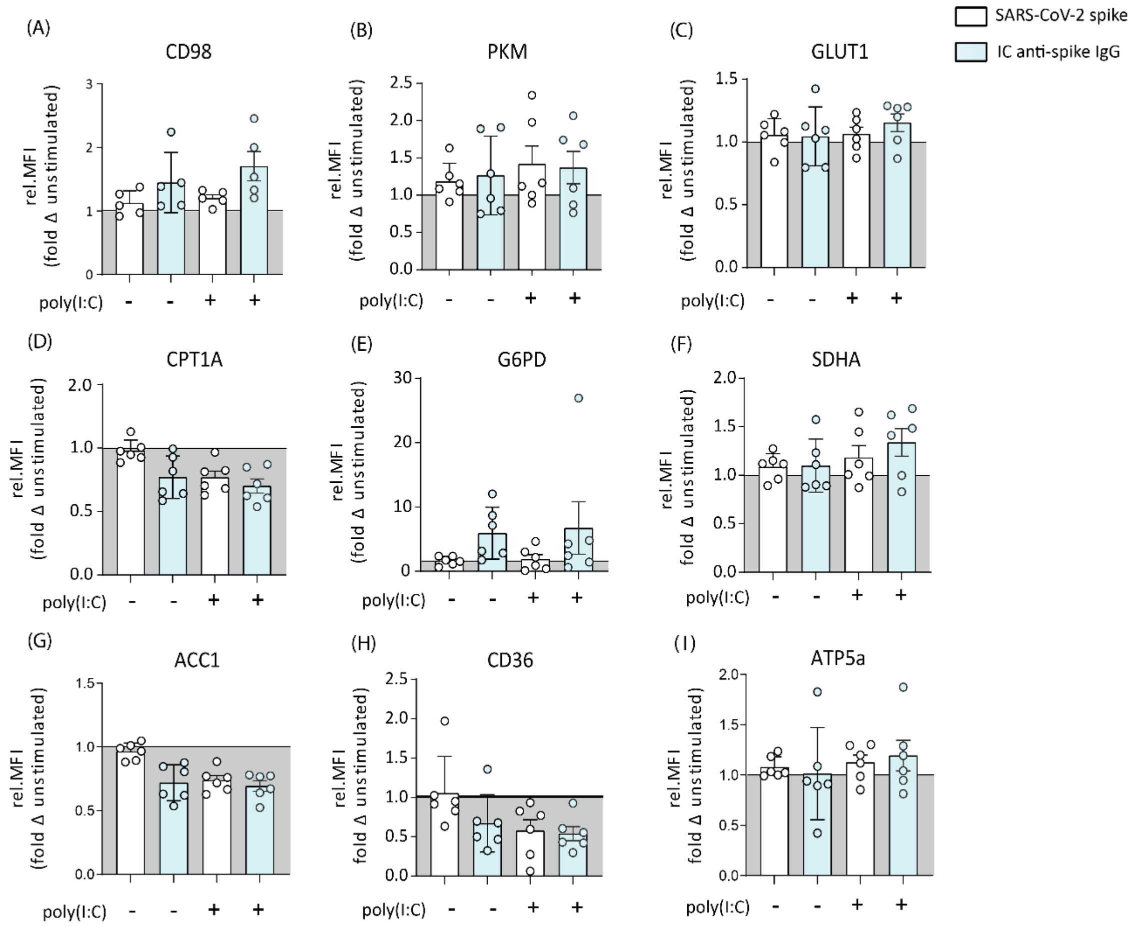

Figure S2. Anti-spike IgG induced change in metabolic enzymes after 24 h stimulation. (A-I) Changes in relative MFI of metabolic enzymes of human macrophages after 24 h stimulation with ICs of recombinant with or without viral stimulus poly(I:C). Data points from individual donors (n = 5, mean + SD).

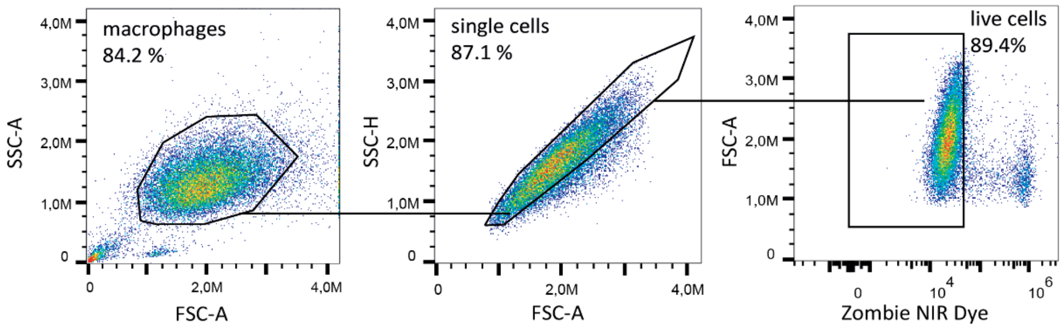

Figure S3: Gating strategy FACS. We selected human-like alveolar macrophages by applying a forward- and side-scatter gate. From this gate singles cells were selected followed by exclusion of dead cells by a live cell gate (Zombie NIR negative population).

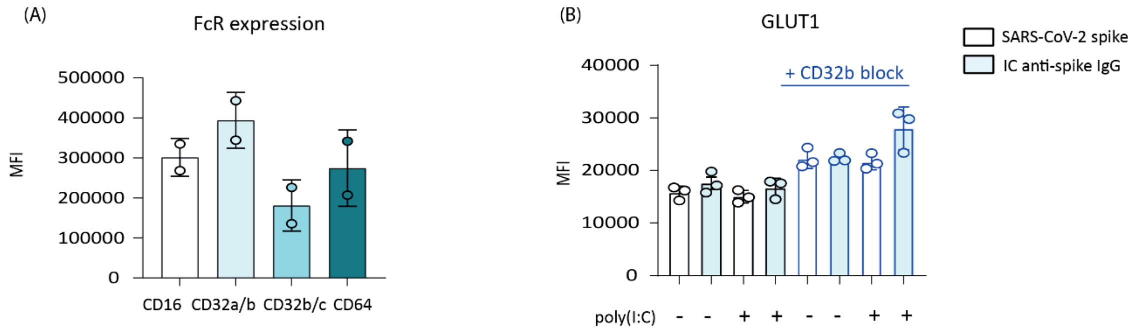

Figure S4. FcγR expression levels on human alveolar-like macrophages and effect of FcγRIIb blockage on anti-spike IgG IC induced changes in GLUT1 expression. (A) Human alveolar-like macrophages were harvested and stained with CD16-PE, CD32a/b-PerCP, CD32b/c-APC-A700, CD64-ECD to determine FcR expression levels. Representative data from n = 2 individual experiments (mean + SD). (B) Glut1 expression of alveolar-like macrophages treated with FcRIIb blocking antibody (blue) compared to untreated cells (black) . Samples were stimulated for 24 h stimulation with ICs of recombinant anti-spike IgG with or without viral stimulus poly(I:C) (n = 3, mean + SD).

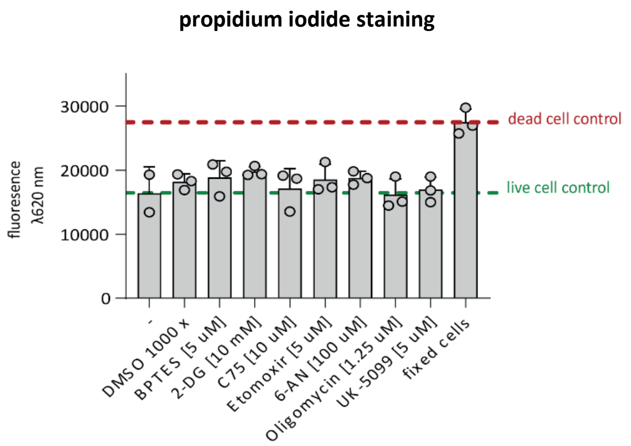

Figure S5. Effect of metabolic inhibitors on cell viability of human alveolar-like macrophages. Human macrophages were incubated with the indicated inhibitor concentration under culture conditions for 24 h. Afterwards, extracellular DNA content was determined via PI staining. Increased extracellular DNA content leads to elevated fluorescence at 620 nm. Representative data from n = 4 individual experiments (mean + SD).
